# Supplementary material for: Distinct dynamics of social motivation drive differential social behavior in laboratory rat and mouse strains
Source: Nat Commun. 2020 Nov 20;11:5908. doi: 10.1038/s41467-020-19569-0 (PMC7679456; doi:10.1038/s41467-020-19569-0)
Supplement: Supplementary file 1 — Supplementary Information [file 41467_2020_19569_MOESM1_ESM.pdf]

Supplementary Information for Netser et al., "Distinct dynamics of social motivation drive differential social behavior in laboratory rat and mouse strains"

List of supplementary figures:

Supplementary Figure 1. Distributions of investigation bout durations during the SP and SNP tests

Supplementary Figure 2. Distinct behavioral dynamics of female C57BL/6J mice and SD rats in the social preference (SP) test

Supplementary Figure 3. Analysis of SP behavior of BALB/c mice

Supplementary Figure 4. SP/SNP paradigms of C57BL/6J mice and SD rats and SNP results of the various strains.

Supplementary Figure 5. Similar behavioral dynamics of C57BL/6J mice and SD rats in the sex preference (SxP) test.

Supplementary Figure 6. Free social interactions in SD rats and C57BL/6J mice.

Supplementary Figure 8. Significant induction of c-Fos expression following 5, but not 2 minutes of social interaction in the NAc shell and MeA of C57BL/6J mice.

Supplementary Figure 9. Comparison between the model and experimental results for SP test in mice.

Supplementary Figure 10. Comparison between the model and experimental results for SNP test in mice.

Supplementary Figure 11. Comparison between the model and experimental results for SP test in rats.

Supplementary Figure 12. Comparison between the model and experimental results for SNP test in rats.

Supplementary Figures

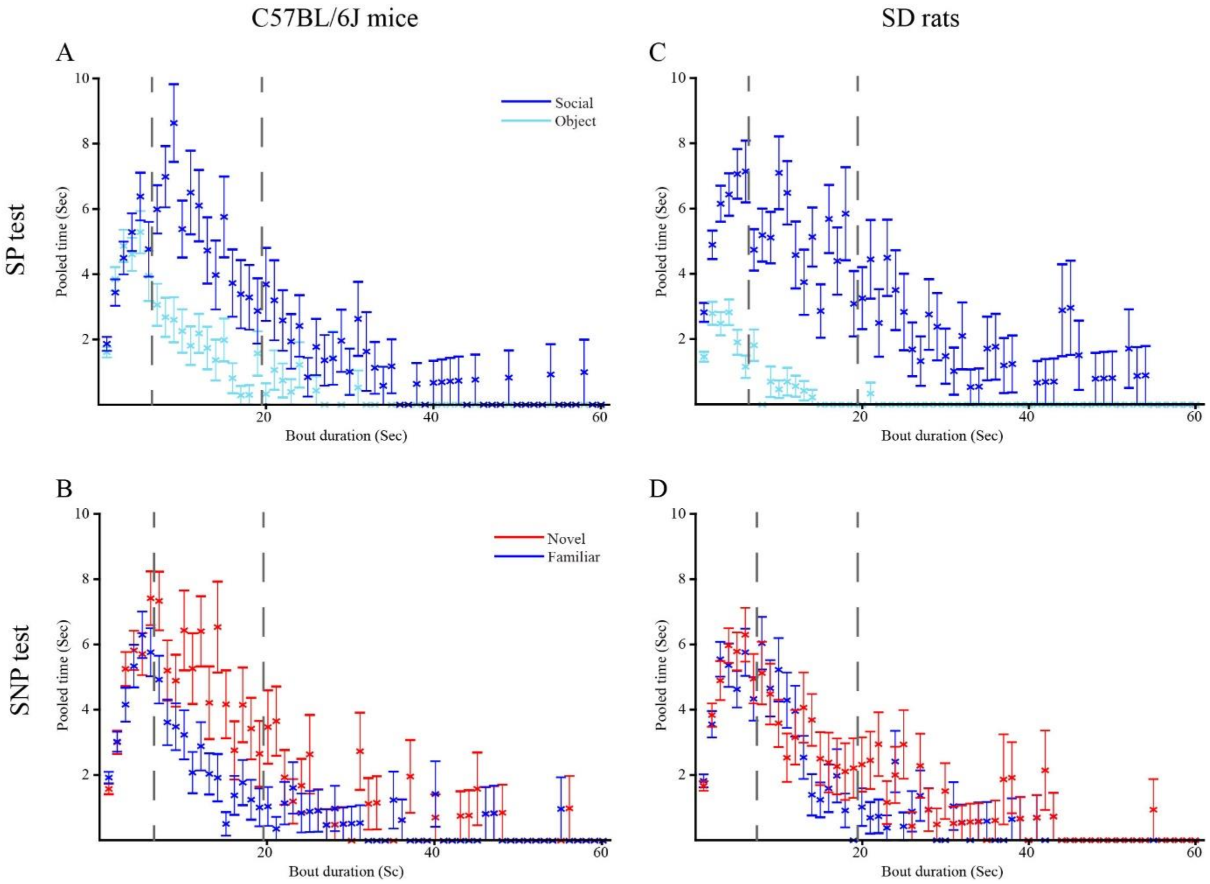

Supplementary Figure 1. Distributions of investigation bout durations during the SP and SNP tests.

A) Superimposed distributions of investigation bout duration for C57BL/6J mice (n=58) during the SP test for social (blue) or object (light blue) stimuli. Dashed lines represent the borders between the populations of short (<6 sec), intermediate (>6 sec, < 19 sec) and long (>19 sec) bouts. Both borders mark clear deeps in the distributions of social and object investigation bouts thus define separated bout populations.

B) As in A, for the SNP test with novel (red) and familiar (Blue) social stimuli. Dashed lines represent the same values as in A, although the distinct populations of investigation bouts are not well-defined in the SNP test.

C) As in A, for SD rats (n=60).

D) As in B, for rats.

All data are presented as Mean ± SEM. Source data are provided as a Source Data file.

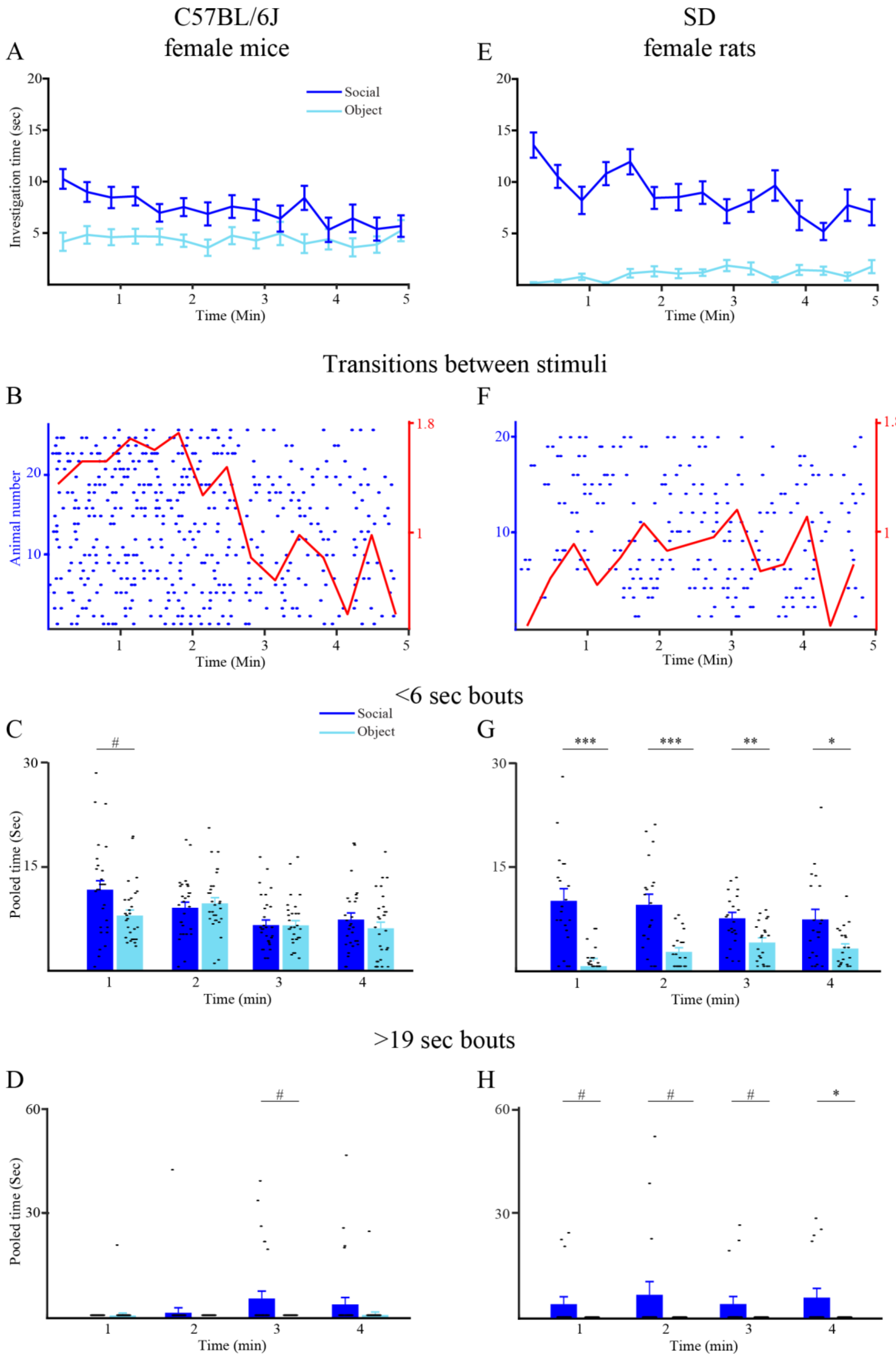

Supplementary Figure 2. Distinct behavioral dynamics of female C57BL/6J mice and SD rats in the social preference (SP) test.

A) Mean investigation time of social and object stimuli, averaged in 20 s bins (n=26 female C57BL/6J mice) across time during the 5-min long SP test.

B) Transitions between the two stimuli, made by subject mice across time during the test. Each punctum denotes the beginning of investigation of a new stimulus, and each row represent a single subject. The mean rate (using 20-sec bins) is denoted by the red line (right red Y-axis).

C) Mean pooled time of short investigation bouts (<6 sec) across time during the SP test (using 1-min bins, last minute excluded; see Methods). 2-way repeated ANOVA, stimuli - $F_{1,25}$=1.647, p=0.211; time - $F_{3,75}$=8.597, p<0.001; stimuli x time - $F_{3,75}$=1.589, p=0.199; #p=0.07, *post hoc* 2-tail paired t-test following main effect.

D) As in C, when extended bouts (>19 sec) are considered. 2-way repeated ANOVA, stimuli - $F_{1,25}$=6.675, p=0.016; time - $F_{3,75}$=1.411, p=0.253; stimuli x time - $F_{3,75}$=1.422, p=0.243. #p=0.051, *post hoc* 2-tail paired t-test following main effect. Black lines at the bottom of the bars represent data points with a value of zero.

E) As in A, for SD rats (n=20 female rats).

F) As in B, for SD rats.

G) As in C, for SD rats. 2-way repeated ANOVA, stimuli - $F_{1,19}$=63.269, p<0.001; time - $F_{3,57}$=0.149, p=0.881; stimuli x time - $F_{3,57}$=2.505, p=0.068. *p<0.05, **p<0.01, ***p<0.001, *post hoc* 2-tail paired t-test following main effect.

H) As in D, for SD rats. 2-way repeated ANOVA, stimuli - $F_{1,19}$=12.543, p=0.002; time - $F_{3,57}$=0.239, p=0.778; stimuli x time - $F_{3,57}$=0.239, p=0.778; #p<0.1, *p<0.05, *post hoc* 2-tail paired t-test following main effect. Black lines at the bottom of the bars represent data points with a value of zero.

All error bars represent SEM. Source data are provided as a Source Data file.

Supplementary Figure 3. Analysis of SP behavior of BALB/c mice.

A) Mean investigation time of social and object stimuli, averaged in 20-sec bins for BALB/c mice (n=21) across time during the 5-min long SP test.

B) Transitions between the two stimuli, made by BALB/c mice across time during the test. Each punctum denotes the beginning of investigation of a new stimulus, and each row represent a single subject. The mean rate (using 20-sec bins) is denoted by the red line (right red Y-axis).

C) Mean pooled time of short investigation bouts (<6 sec) made by BALB/c mice across time during the SP test (using 1-min bins, last minute excluded; see Methods).

D) As in C, when extended bouts (>19 sec) are considered. *p<0.05, **p<0.01, ***p<0.001, *post hoc* 2-tail t-test following main effect. Black lines at the bottom of the bars represent data points with a value of zero.

E) Comparison of mean RDI values between BALB/c (n=21, black bars) and C57BL/6J (n=58, grey bars) mice tested with the SP test.

F) Comparison of mean transition rate during the first minute of the SP test, between BALB/c (black bars) and C57BL/6J (grey bars) mice. **p<0.01, 2-tail Mann-Whitney test.

G) Comparison of mean bout duration during the first minute of the SP test, between BALB/c (black bars) and C57BL/6J (grey bars) mice. *p<0.05, 2-tail Mann-Whitney test.

All error bars represent SEM. Source data are provided as a Source Data file.

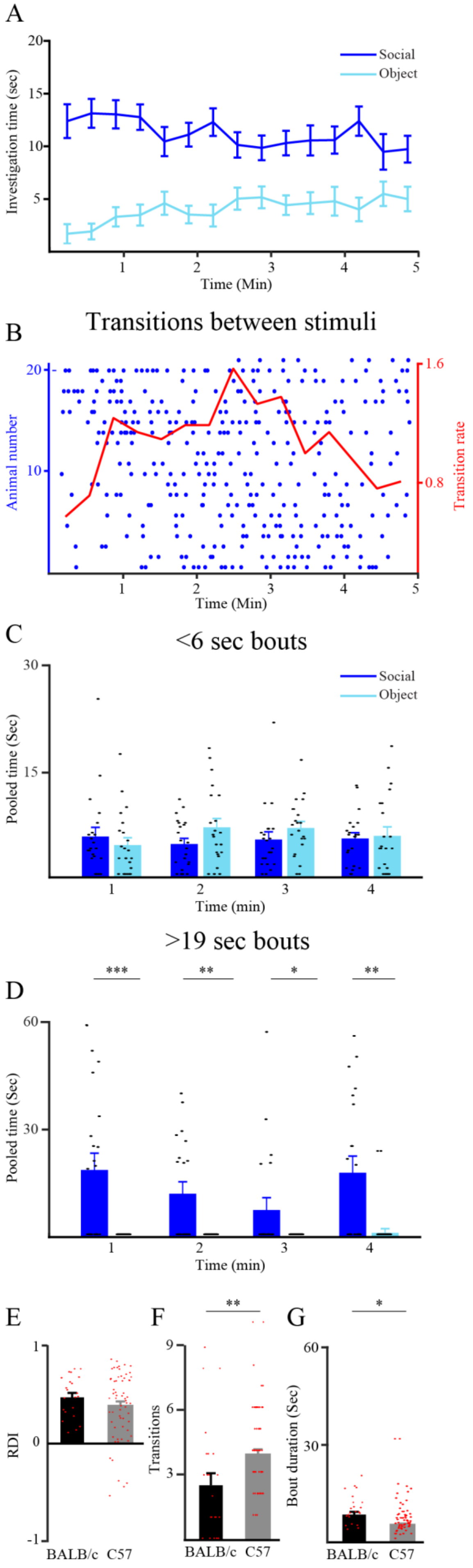

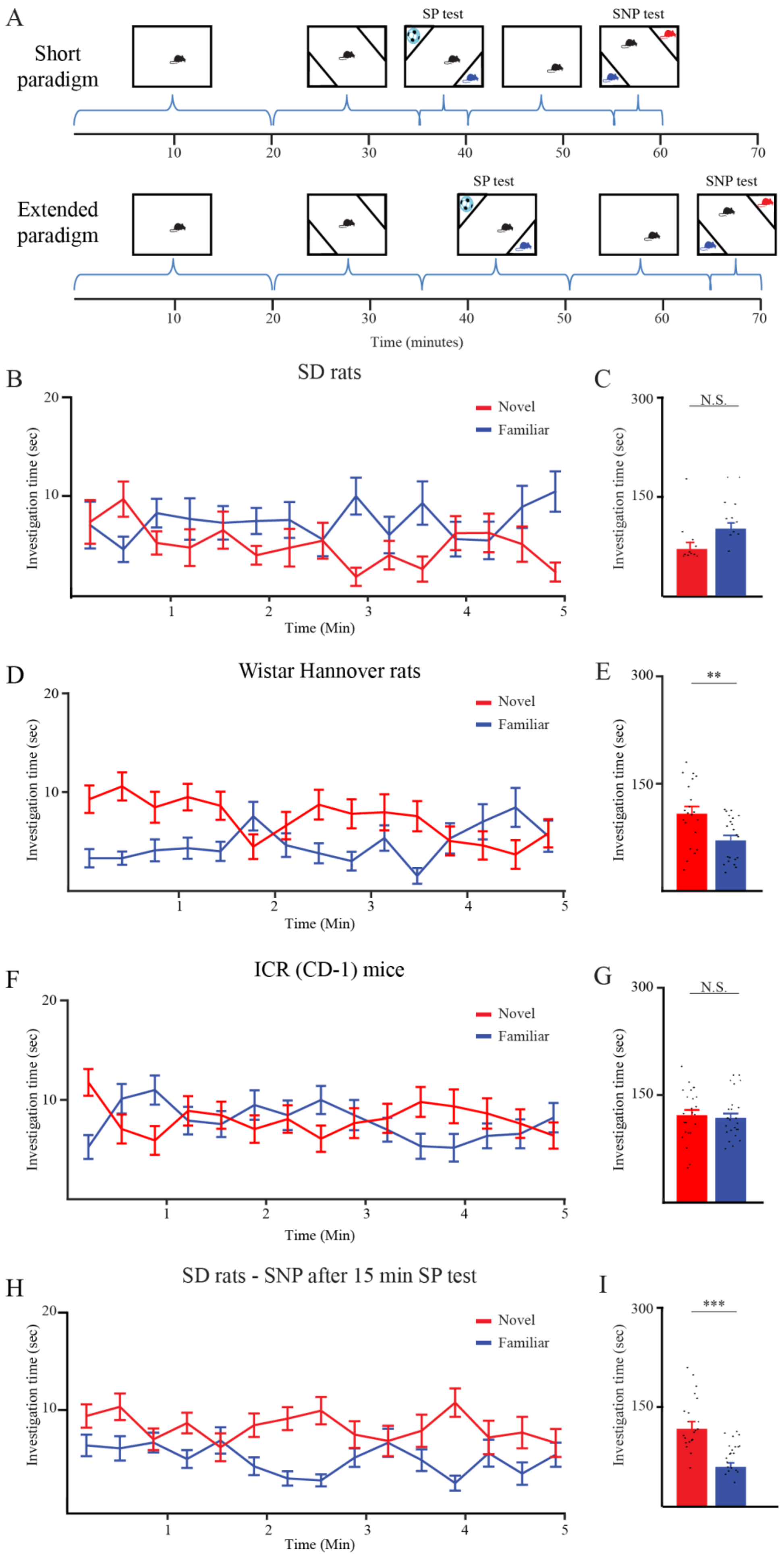
A
Short paradigm
SP test
SNP test
Extended paradigm
Time (minutes)
B
SD rats
Novel
Familiar
Investigation time (sec)
Time (Min)
C
N.S.
D
Wistar Hannover rats
E
**
F
ICR (CD-1) mice
G
N.S.
H
SD rats - SNP after 15 min SP test
I
***

Supplementary Figure 4. SP/SNP paradigms of C57BL/6J mice and SD rats and SNP results of the various strains.

A)

Short Paradigm - timeline of the short SP/SNP paradigm used for mice with each stage represented by a boxed cartoon while the timeline (in minutes) is depicted below.

Extended paradigm – timeline for the extended SP/SNP paradigm used for SD rats, after they didn't show social novelty preference when performing the short paradigm. Note that the only difference between the paradigms is the duration of the SP test (15 min instead of 5 min).

B-C) Results of SNP test of SD rats using the short paradigm (n=10). p=0.086, 2-tail paired t-test.

D-E) Results of SNP test of Wistar Hannover rats using the short paradigm (n=20). Note that unlike SD rats, Wistar Hannover rats did show clear social novelty preference (**p=0.007, 2-tail paired t-test) in the short paradigm, like C57BL/6J mice.

F-G) Results of SNP test of ICR mice using the short paradigm (n=24). Note that unlike C57BL/6J mice, ICR mice showed no social novelty preference (p=0.751, 2-tail paired t-test) in the short paradigm, similarly to SD rats.

H-I) Results of SNP test conducted with SD rats using the extended paradigm (n=20). Note the significant social novelty preference (***p<0.001, 2-tail paired t-test) exhibited by SD rats.

All error bars represent SEM. Source data are provided as a Source Data file.

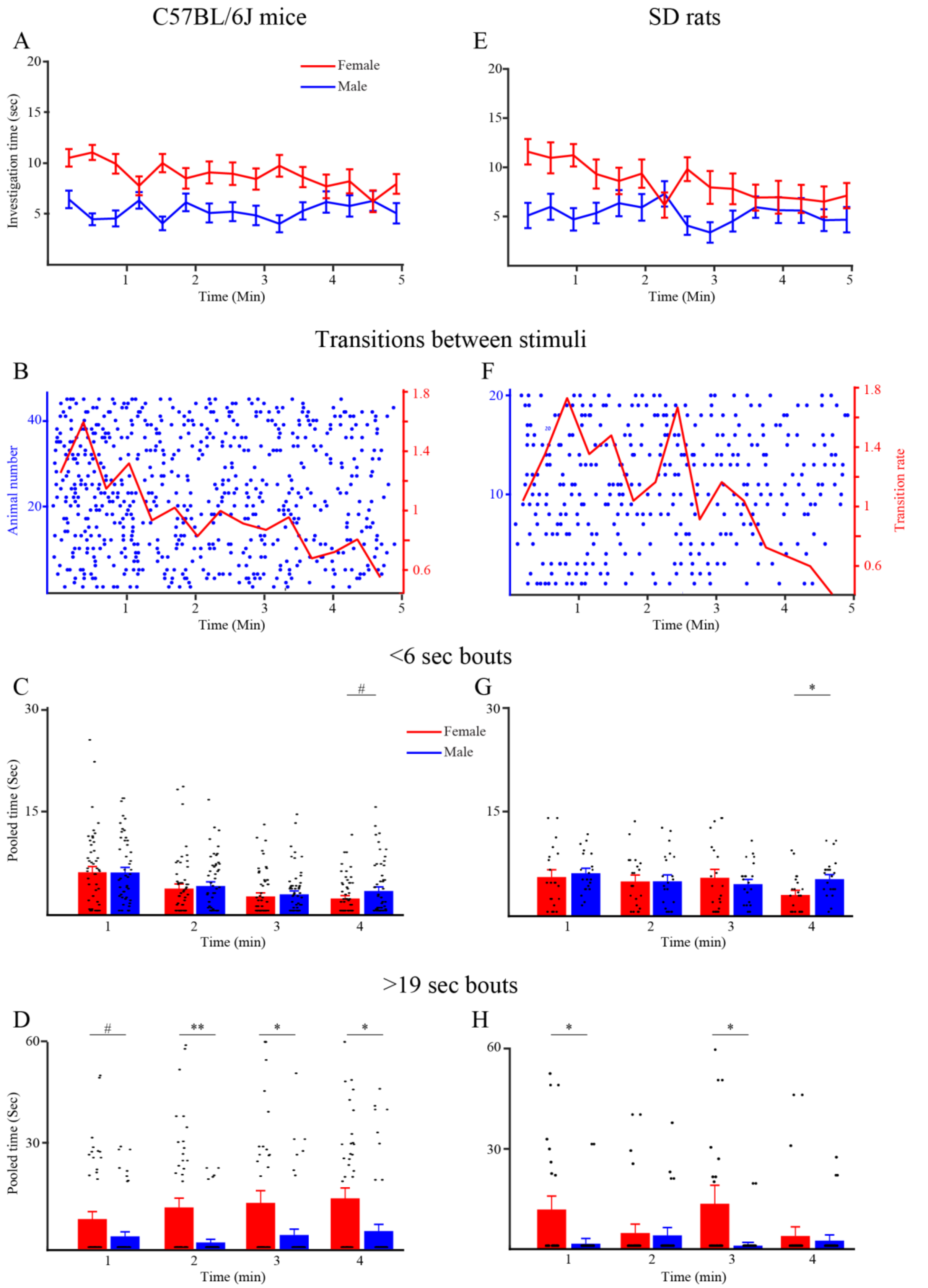

Supplementary Figure 5. Similar behavioral dynamics of C57BL/6J mice and SD rats in the sex preference (SxP) test.

A) Mean investigation time of female and male social stimuli, averaged in 20 s bins (n=45 male C57BL/6J mice) across time during the 5-min long SxP test.

B) Transitions between the two stimuli, made by subject mice across time during the test. Each punctum denotes the beginning of investigation of a new stimulus, and each row represent a single subject. The mean rate (using 20-sec bins) is denoted by the red line (right red Y-axis).

C) Mean pooled time of short investigation bouts (<6 sec) across time during the SxP test (using 1-min bins, last minute excluded; see Methods). 2-way repeated ANOVA, stimuli - $F_{1,44}$=1.392, p=0.244; time - $F_{3,132}$=11.660, p<0.001; stimuli x time - $F_{3,132}$=0.390, p=0.761. #p=0.094, *post hoc* 2-tail paired t-test following main effect.

D) As in C, when extended bouts (>19 sec) are considered. 2-way repeated ANOVA, stimuli - $F_{1,44}$=16.221, p<0.001; time - $F_{3,132}$=1.441, p=0.234; stimuli x time - $F_{3,132}$=0.573, p=0.634. #p=0.056, *p<0.05, **p<0.01, *post hoc* 2-tail paired t-test following main effect. Black lines at the bottom of the bars represent data points with a value of zero.

E) As in A, for SD rats (n=20 male rats).

F) As in B, for SD rats.

G) As in C, for SD rats. 2-way repeated ANOVA, stimuli - $F_{1,19}$=0.624, p<0.439; time - $F_{3,57}$=1.281, p=0.290; stimuli x time - $F_{3,57}$=1.372, p=0.261.

H) As in D, for SD rats. 2-way repeated ANOVA, stimuli - $F_{1,19}$=4.332, p=0.051; time - $F_{3,57}$=1.039, p=0.382; stimuli x time - $F_{3,57}$=2.494, p=0.069. *p<0.05, *post hoc* 2-tail paired t-test following main effect. Black lines at the bottom of the bars represent data points with a value of zero.

All error bars represent SEM. Source data are provided as a Source Data file.

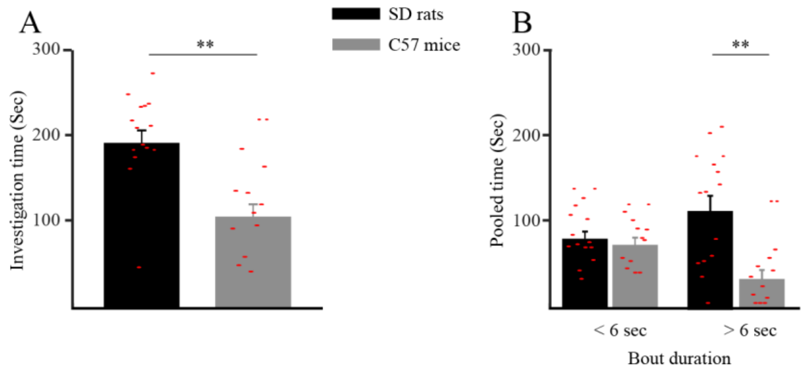

Supplementary Figure 6. Free social interactions in SD rats and C57BL/6J mice.

A) Mean interaction time for SD rats (n=14) and C5BL/6J mice (n=12) during a 5 min free interaction test. **p<0.01, 2-tail independent t-test.

B) As in A, separately analyzed for bouts of interaction which are shorter (left) or longer (right) than 6 sec. As apparent, the difference between the strains stems from the long bouts. Mixed model ANOVA, time x strain - $F_{1,24}$=6.444, p=0.018, **p<0.01, *post hoc* 2-tail t-test following main effect in test.

All error bars represent SEM. Source data are provided as a Source Data file.

Supplementary Figure 7. Analysis of c-Fos induction in the VTA following social encounter.

A) Representative images of c-Fos immunostaining in the VTA, for example C57BL/6J mouse (left) and SD rat (right) control animals that did not encounter social stimulus in the arena (above) and from similar animals that did encounter a social stimulus for free interactions of two minutes (below).

B) Mean values c-Fos positive cells in the VTA for C57BL/6J mice (n=4/group), with (light blue) and without (grey) social interaction.

C) As in B, for SD rats (n=4/group). *p<0.001, 2-tail independent t-test.

All error bars represent SEM. Source data are provided as a Source Data file.

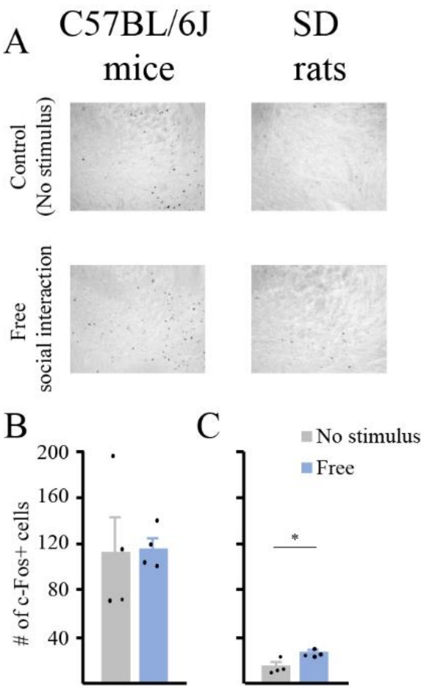

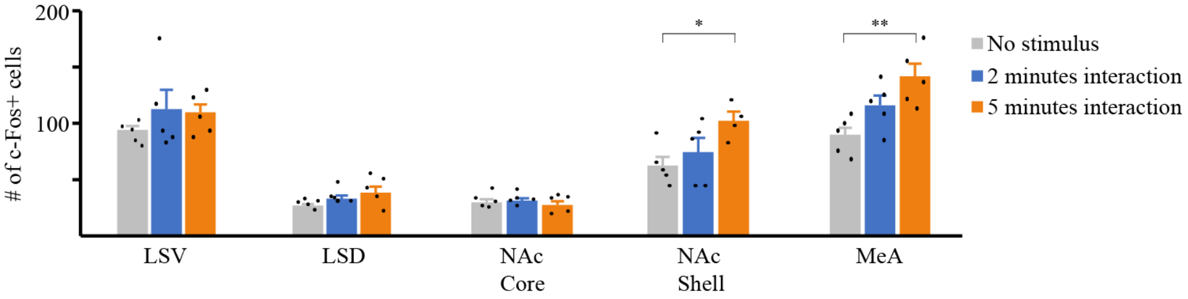

Supplementary Figure 8. Significant induction of c-Fos expression following 5, but not 2 minutes of social interaction in the NAc shell and MeA of C57BL/6J mice.

*p<0.05, **p<0.01, 2-tail independent t-test following main effect in one-way ANOVA test. n=5 animals/group.

All error bars represent SEM. Source data are provided as a Source Data file.

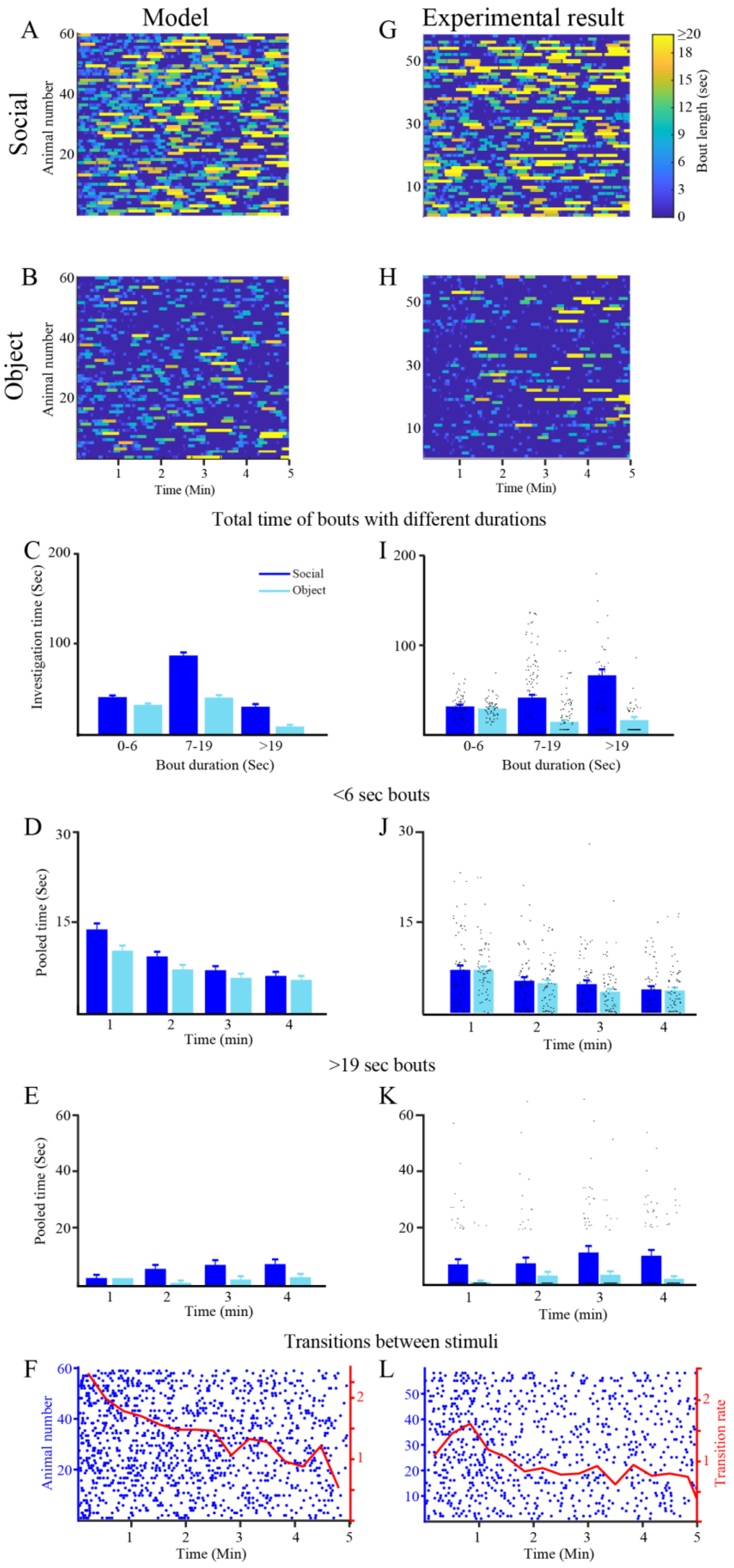

Supplementary Figure 9. Comparison between the model and experimental results for SP test in mice.

A) Heat map of investigation bouts towards the social stimulus of a modeled population of mice (n=60) during the SP test.

B) Same as in A, for the object stimulus.

C) Mean values of investigation time of the modeled population shown in A-B, for the various categories of bout duration.

D) Mean values of investigation time during short (<6 sec) bouts across the first 4 minutes of the test.

E) Same as in D, for long (>19 sec) bouts

F) The transitions along the time course of the experiment. The red line represents the mean transition rate.

G-L) Same as A-F, for the experimental results (n=58 mice). Black lines at the bottom of the bars represent data points with a value of zero.

All error bars represent SEM. Source data are provided as a Source Data file.

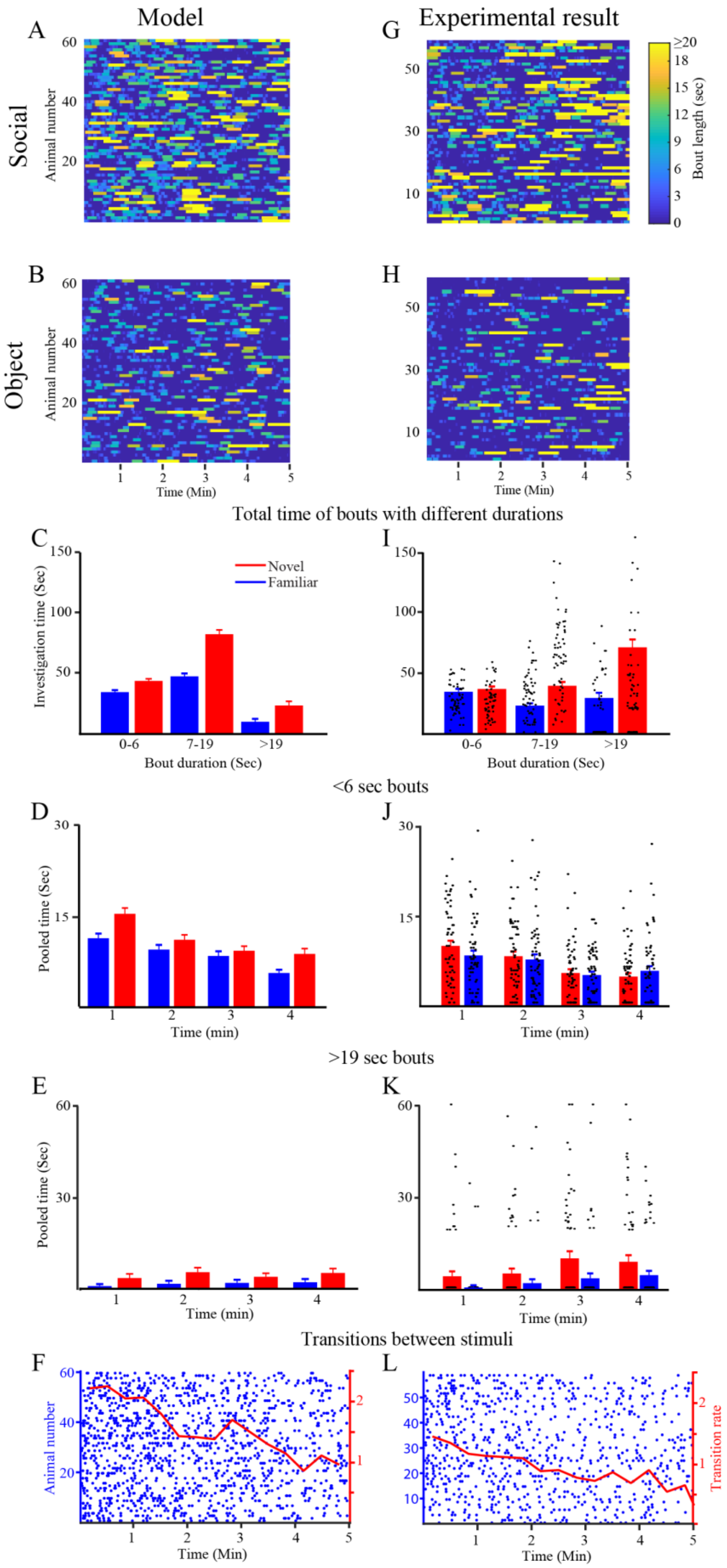

Supplementary Figure 10. Comparison between the model and experimental results for SNP test in mice.

A) Heat map of investigation bouts towards the novel social stimulus of modeled population of mice (n=60) during the SNP test.

B) Same as in A, for the familiar social stimulus.

C) Mean values of investigation time of the modeled population shown in A-B, for the various categories of bout duration.

D) Mean values of the investigation time during short (<6 sec) bouts across the first 4 minutes of the test.

E) Same as in D, for long (>19 sec) bouts

F) The transitions along the time course of the experiment. The red line represents the mean transition rate.

G-L) Same as A-F, for the experimental results (n=58 mice). Black lines at the bottom of the bars represent data points with a value of zero.

All error bars represent SEM. Source data are provided as a Source Data file.

Supplementary Figure 11. Comparison between the model and experimental results for SP test in rats.

A) Heat map of investigation bouts towards the social stimulus of modeled population of rats (n=60) during the SP test.

B) Same as in A, for the object stimulus.

C) Mean values of investigation time of the modeled population shown in A-B, for the various categories of bout duration.

D) Mean values of the investigation time during short (<6 sec) bouts across the first 4 minutes of the test.

E) Same as in D, for long (>19 sec) bouts

F) The transitions along the time course of the experiment. The red line represents the mean transition rate.

G-L) Same as A-F, for the experimental results (n=60 rats). Black lines at the bottom of the bars represent data points with a value of zero.

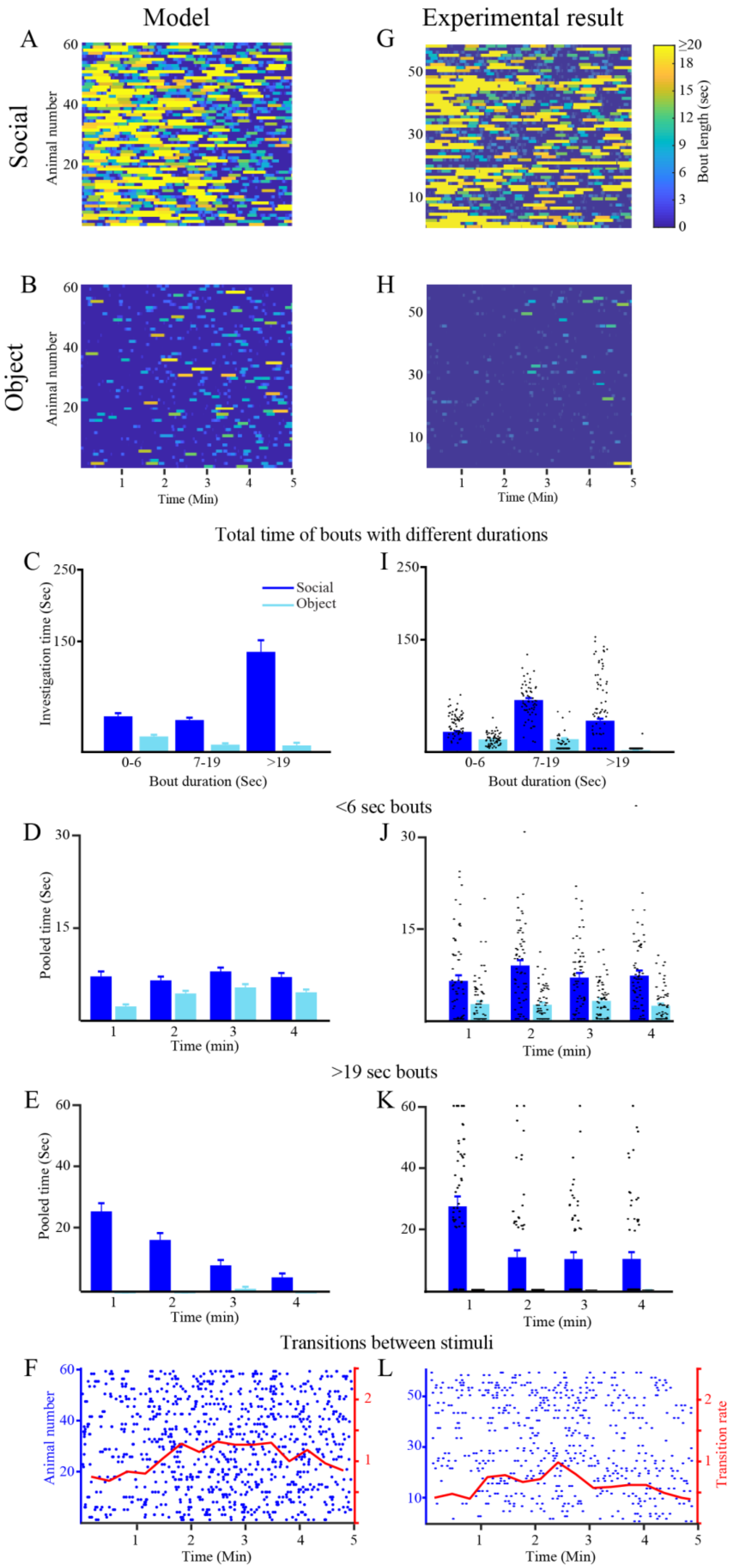

All error bars represent SEM. Source data are provided as a Source Data file.

Supplementary Figure 12. Comparison between the model and experimental results for SNP test in rats.

A) Heat map of investigation bouts towards the novel social stimulus of modeled population of rats (n=60) during the SNP test.

B) Same as in A, for the familiar social stimulus.

C) Mean values of investigation time of the modeled population shown in A-B, for the various categories of bout duration.

D) Mean values of the investigation time during short (<6 sec) bouts across the first 4 minutes of the test.

E) Same as in D, for long (>19 sec) bouts

F) The transitions along the time course of the experiment. The red line represents the mean transition rate.

G-L) Same as A-F, for the experimental results (n=60 rats). Black lines at the bottom of the bars represent data points with a value of zero.

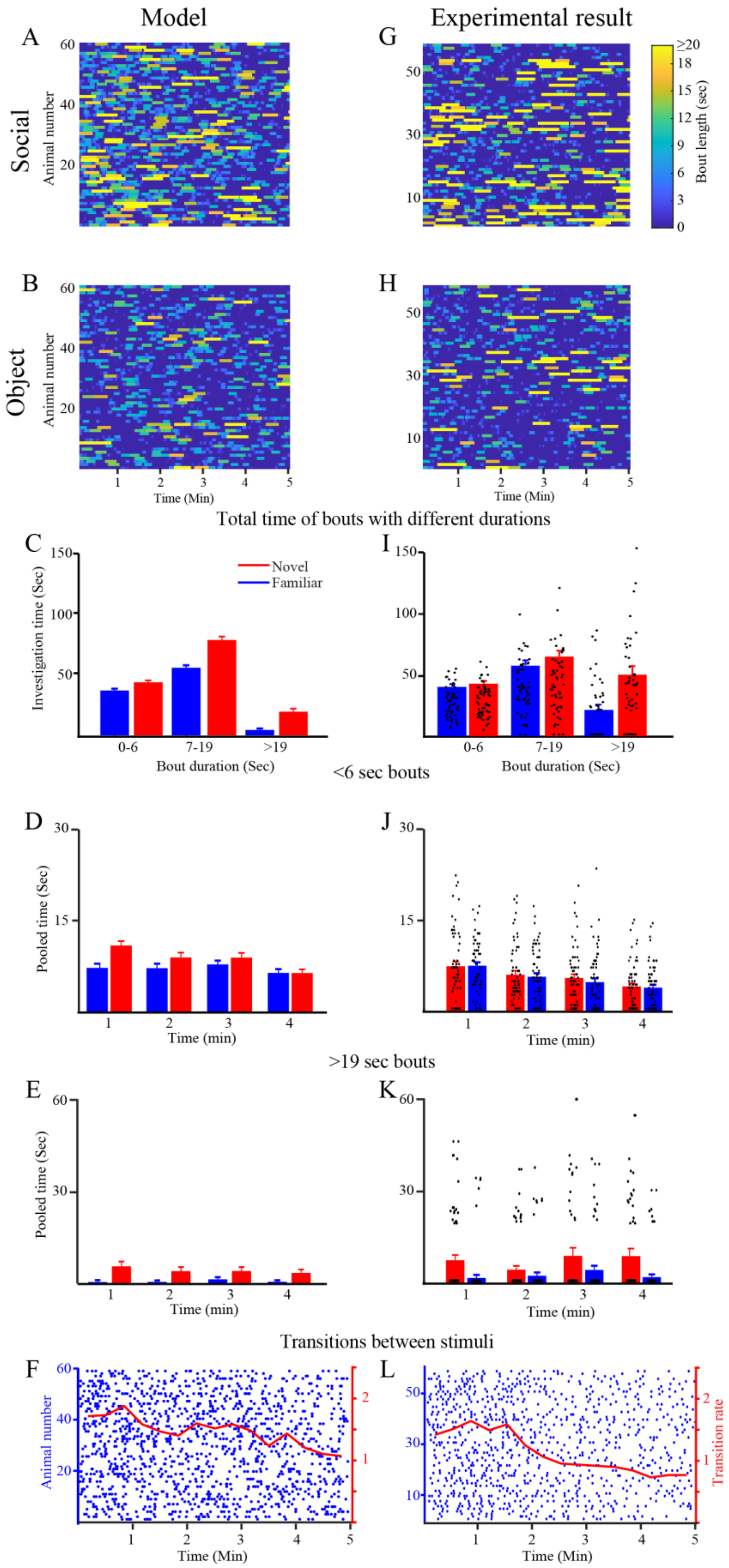

All error bars represent SEM. Source data are provided as a Source Data file.
