# Supplementary material for: Distinct dynamics of social motivation drive differential social behavior in laboratory rat and mouse strains
Source: Nat Commun. 2020 Nov 20;11:5908. doi: 10.1038/s41467-020-19569-0 (PMC7679456; doi:10.1038/s41467-020-19569-0)
Supplement: Supplementary file 3 — Reporting Summary [file 41467_2020_19569_MOESM3_ESM.pdf]

nature research

Corresponding author(s): Shlomo Wagner
Last updated by author(s): 25/08/2020

Reporting Summary

Nature Research wishes to improve the reproducibility of the work that we publish. This form provides structure for consistency and transparency in reporting. For further information on Nature Research policies, see Authors & Referees and the Editorial Policy Checklist.

Statistics

For all statistical analyses, confirm that the following items are present in the figure legend, table legend, main text, or Methods section.

n/a	Confirmed	
☐	☒	The exact sample size (n) for each experimental group/condition, given as a discrete number and unit of measurement
☐	☒	A statement on whether measurements were taken from distinct samples or whether the same sample was measured repeatedly
☐	☒	The statistical test(s) used AND whether they are one- or two-sided<br>*Only common tests should be described solely by name; describe more complex techniques in the Methods section.*
☐	☒	A description of all covariates tested
☐	☒	A description of any assumptions or corrections, such as tests of normality and adjustment for multiple comparisons
☐	☒	A full description of the statistical parameters including central tendency (e.g. means) or other basic estimates (e.g. regression coefficient) AND variation (e.g. standard deviation) or associated estimates of uncertainty (e.g. confidence intervals)
☐	☒	For null hypothesis testing, the test statistic (e.g. F, t, r) with confidence intervals, effect sizes, degrees of freedom and P value noted<br>*Give P values as exact values whenever suitable.*
☐	☒	For Bayesian analysis, information on the choice of priors and Markov chain Monte Carlo settings
☒	☐	For hierarchical and complex designs, identification of the appropriate level for tests and full reporting of outcomes
☐	☒	Estimates of effect sizes (e.g. Cohen's d, Pearson's r), indicating how they were calculated

Our web collection on statistics for biologists contains articles on many of the points above.

Software and code

Policy information about availability of computer code

Data collection	Video acquisition: FlyCapture2, Point Grey (Ver 2.7.3.18).<br>Social stimuli movements: Intan RHD2000 evaluation board and software, connected to a custom-made electrical circuit described in Figure 5.
Data analysis	MATLAB (MathWorks, Natick, MA, USA) versions 2017a-2019b was used for video and movement analysis as well as computational modeling.<br>Video: "TrackRodent"custom-made code, available at GitHub [https://github.com/shainetser/TrackRodent].<br>Stimulus's movement: custom-made code, available at GitHub [https://github.com/shainetser/PiezoElectricSensors_Code]<br>Computational Model: custom-made code publicly available at GitHub [https://github.com/shainetser/Computational-model-of-social-preference-behavior-]<br>c-Foc expression: Fiji free software (Ver. 20160205) [http://www.imagej.net/Fiji].<br>Statistics: SPSS 21.0.

For manuscripts utilizing custom algorithms or software that are central to the research but not yet described in published literature, software must be made available to editors/reviewers. We strongly encourage code deposition in a community repository (e.g. GitHub). See the Nature Research guidelines for submitting code & software for further information.

Data

Policy information about availability of data

All manuscripts must include a data availability statement. This statement should provide the following information, where applicable:

- Accession codes, unique identifiers, or web links for publicly available datasets
- A list of figures that have associated raw data
- A description of any restrictions on data availability

The data that support the findings of this study are available from the corresponding author upon reasonable request.

Field-specific reporting

Please select the one below that is the best fit for your research. If you are not sure, read the appropriate sections before making your selection.

☒ Life sciences ☐ Behavioural & social sciences ☐ Ecological, evolutionary & environmental sciences

For a reference copy of the document with all sections, see nature.com/documents/nr-reporting-summary-flat.pdf

Life sciences study design

All studies must disclose on these points even when the disclosure is negative.

Sample size	Behavioral experiments included groups of: 58,28, 45 and 16 male C57BL/6J mice and 60,24, 20 and 8 male SD rats (SP+NP,Social stimuli movements' measurements, SxP and social vs Food preference, respectively); 26 and 20 female C57BL/6J and SD rats, respectively; 21, 24 and 20 BALB/c mice, ICR mice and Wistar Hannover rats, respectively. Our previous publication (PMID: 29026510) with similar type of experiments and data analysis showed that sample sizes of five and eight animals are required for the SP and SNP tests, respectively (α=0.05, power=0.8). Thus, our current sample sizes for the similar experiments performed here are sufficient and above. We have done our best to make the sample size of rats and mice as equal as possible.
Data exclusions	No data or animals were excluded.
Replication	In all behavioral experiments large groups were sampled (see sample sizes above). However, all statistics were also performed on 2-3 sub-groups of 10-20 animals each (finally joined to the full sample) and were found to show significance or a similar trend, thus validating one another. As for c-Fos experiments, mice experiments were repeated in a second group, with no significant change in the results. The social vs. food experiment was conducted with only one group of rats, as the results were very clear.
Randomization	All experiments were fully randomized between subjects and stimuli animals from the same sex, strains or species.
Blinding	Behavioral comparison between distinct rat and mice strains cannot be done blindly at the data collection stage, as these strains are clearly distinct from each other. However, all data collection was fully automated, such that there was no way for the experimentalists to get involved in it. Moreover, the experimentalists were blinded during the analysis, which was fully computerized. Furthermore, the experimentalists were undergraduate students hired for the project and they were not aware of the experimental design or hypothesis.

Reporting for specific materials, systems and methods

We require information from authors about some types of materials, experimental systems and methods used in many studies. Here, indicate whether each material, system or method listed is relevant to your study. If you are not sure if a list item applies to your research, read the appropriate section before selecting a response.

Materials & experimental systems

n/a	Involved in the study	
☐	☒	Antibodies
☒	☐	Eukaryotic cell lines
☒	☐	Palaeontology
☐	☒	Animals and other organisms
☒	☐	Human research participants
☒	☐	Clinical data

Methods

n/a	Involved in the study	
☒	☐	ChIP-seq
☒	☐	Flow cytometry
☒	☐	MRI-based neuroimaging

Antibodies

Antibodies used

PRIMARY ANTIBODY:
Name: c-Fos (9F6) Rabbit mAb
Catalog number: #2250
Lot number: 10
Dilution: 1:500
Supplier: Cell Signaling Technology
https://www.cellsignal.de/products/primary-antibodies/c-fos-9f6-rabbit-mab/2250
The c-Fos signals were revealed using a biotinylated secondary antibody and ABC kit (Vector Laboratories).
SECONDARY ANTIBODY:
Name: Goat Anti-Rabbit IgG Antibody (H+L), Biotinylated
Catalog number: BA-1000
Lot number: Z0619
Dilution: 1:500
Supplier: Vector Laboratories

https://vectorlabs.com/biotinylated-goat-anti-rabbit-igg-antibody.html

Validation

Manufacturer statement: This antibody has been validated using SimpleChIP® Enzymatic Chromatin IP Kits. It is cross-reactive to human, mouse, rat, hamster, bovine and pig and it is applicable to western blotting, immunofluorescence (immunocytochemistry), flow cytometry and chromatin IP.
Relevant citation: https://pubmed.ncbi.nlm.nih.gov/32369018/.

Animals and other organisms

Policy information about studies involving animals; ARRIVE guidelines recommended for reporting animal research

Laboratory animals

All animals were kept in the animal facility of the University of Haifa under veterinary supervision, with ad libitum access to food (standard chow diet, Envigo RMS, Israel) and water. Mice subjects were naïve C57BL/6J, BALB/c or ICR (CD-1) adult male or female mice (10-15 weeks), commercially obtained (Envigo, Israel) and housed in groups of 2-5 per cage. Mice stimuli were in-house grown C57BL/6J, BALB/c or ICR juvenile male or female mice (21-30 days old), besides the sex preference test where stimuli were adult female and male C57BL/6J mice (8-12 weeks old). Mice were kept on a 12 h light/12 h dark cycle, lights on at 7 PM. Rats subjects were Sprague Dawley (SD) or Wistar Hannover male or female rats (10-15 weeks), commercially obtained (Envigo, Israel) or grown in-house. Rat stimuli were in-house grown SD or Wistar Hannover juvenile male or female rats (21-30 days old) commercially obtained (Envigo, Israel), besides the sex preference test where stimuli were adult female and male SD rats (8-12 weeks old). Rats were kept in groups of 2-5 animals per cage, in a 12 h light/12 h dark cycle, lights on at 9 PM. Behavioral experiments took place during the dark phase of the animals, under dim red light. All experiments were performed according to the National Institutes of Health guide for the care and use of laboratory animals, and approved by the Institutional Animal Care and Use Committee (IACUC) of the University of Haifa.

Wild animals

None

Field-collected samples

None

Ethics oversight

All experiments were approved by the Institutional Animal Care and Use Committee (IACUC) of the University of Haifa.

Note that full information on the approval of the study protocol must also be provided in the manuscript.
