# Supplementary material for: Distinct dynamics of social motivation drive differential social behavior in laboratory rat and mouse strains
Source: Nat Commun. 2020 Nov 20;11:5908. doi: 10.1038/s41467-020-19569-0 (PMC7679456; doi:10.1038/s41467-020-19569-0)
Supplement: Supplementary file 4 — Description of Additional Supplementary Files [file 41467_2020_19569_MOESM4_ESM.pdf]

Description of Additional Supplementary Files

File Name: Supplementary Movie 1
Description: **Example movie of a C57BL/6J mouse during SP test** The while cross on the body mass center of the mouse changes to green during active exploration of the social stimulus, and to blue during active exploration of the object.

File Name: Supplementary Movie 2
Description: **Example movie of a SD rat during SP test** The red cross on the body mass center of the rat changes to green during active exploration of the social stimulus, and to black during active exploration of the object.

File Name: Supplementary Data 1
Description: **Table of statistical results** This table details the results of all statistical tests conducted for the study, listed according to the relevant figures and supplementary figures.

File Name: Supplementary Data 2
Description: **Code of computational model** This ZIP file contains the MATLAB code for simulating social preference (SP) and social novelty preference (SNP) behavior, as well as a mannual explaining how to use it.
