# Supplementary material for: Distinct dynamics of social motivation drive differential social behavior in laboratory rat and mouse strains
Source: Nat Commun. 2020 Nov 20;11:5908. doi: 10.1038/s41467-020-19569-0 (PMC7679456; doi:10.1038/s41467-020-19569-0)
Supplement: Supplementary file 8 — Supplementary Data 2 [file 41467_2020_19569_MOESM8_ESM.zip › Model code/Model running instruction - 23-3-2020.docx]

**Computational model of social preference behavior**

The code was written in Matlab (2017a). It was tested using on a standard HP-i7 computer (i7-4790 CPU @ 3.60 GHz, 8.0GB RAM, windows 7). Run time on such a computer is about 10 seconds.

Installation and running:

1. **Download the model from the following link:**
2. **Open Matlab and select your working directory to be the model file folder.
   The folder should contain the file – “Show_model_social_sim _015052018.m”.**
3. **Run the model using the following function:**[seq] = Show_model_social_sim_Shai_015052018(params)
   % when the model params are:
   % [anxiety_0, anxiety_tau, reward_0_1, reward_0_2, reward_tau, beta1_reward, beta2_reward, beta1_anxiety, beta2_anxiety, choose_Stillness_score_0, choose_Exploration_score_0];
4. **For SP mice type:
   Open the code of the function “Show_model_social_sim _015052018.m”
   Comment lines 53-54 and uncomment lines 51-52.**[seq] = Show_model_social_sim_Shai_015052018([7.66,10355.62,4.98,4.72,100406.23,1.10,1.12,-0.08,0.71,2.72,5.19]);
5. **For SNP mice type:
   Open the code of the function “Show_model_social_sim _015052018.m”
   Comment lines 53-54 and uncomment lines 51-52.**[seq] = Show_model_social_sim_Shai_015052018([7.66,10355.62,4.7,4.9,100406.23,1.10,1.12,-0.08,0.71,2.72,5.19]);
6. **For SP rats type:
   Open the code of the function “Show_model_social_sim _015052018.m”
   Comment lines 51-52 and uncomment lines 53-54.**[seq] = Show_model_social_sim_Shai_015052018([7.66,10355.62,5.8,4.72,33406.23,1.10,1.12,-0.08,0.71,2.72,5.19]);
7. **For SNP rats type:
   Open the code of the function “Show_model_social_sim _015052018.m”
   Comment lines 51-52 and uncomment lines 53-54.**[seq] = Show_model_social_sim_Shai_015052018([7.66,10355.62,5.17,5.3,33406.23,1.10,1.12,-0.08,0.71,2.72,5.19]);
8. **A Matlab figure should open at the end and hold 8 subplots, similar to supplemental figures 8-11.**
